# Effects of pressure control and pressure support ventilation on ventilator induced lung injury in experimental acute respiratory distress syndrome with intra-abdominal hypertension

**DOI:** 10.1186/2197-425X-3-S1-A806

**Published:** 2015-10-01

**Authors:** CL Santos, RS Santos, L Moraes, CS Samary, NS Felix, PL Fiorio Júnior, MM Morales, MG Abreu, P Pelosi, A Schanaider, PL Silva, PRM Rocco

**Affiliations:** Laboratory of Pulmonary Investigation, Federal University of Rio de Janeiro, Rio de Janeiro, Brazil; Department of Surgery, Faculty of Medicine, Federal University of Rio de Janeiro, Rio de Janeiro, Brazil; D'Or Institute for Research and Education, Hospital Copa D'Or, Rio de Janeiro, Brazil; D'Or Institute for Research and Education, Hospital Caxias D'Or, Duque de Caxias, Brazil; Department of Anesthesiology, Dresden University of Technology, Dresden, Germany; Department of Surgical Sciences and Integrated Diagnostics, University of Genoa, Genoa, Italy

## Introduction

In acute respiratory distress syndrome (ARDS), intra-abdominal hypertension (IAH) increases intra-thoracic pressures, leading atelectasis and deterioration of respiratory mechanics and gas-exchange. The optimal setting of mechanical ventilation (MV) and its impact on respiratory function and ventilator-induced lung injury (VILI) in ARDS associated with IAH needs to be better clarified. Lung-protective MV with low tidal volume (V_T_) and positive end-expiratory pressure (PEEP) has been recommended; however, assisted MV may be a favorable alternative to controlled MV at the early phase of ARDS, since it requires less sedation, no paralysis and is associated with better lung protection, reducing the risk of VILI. We hypothesized that pressure-support ventilation (PSV) improve pulmonary morphofunction and minimize lung injury in ARDS with IAH.

## Objectives

To compare the effects of PSV with protective MV (PCV) on arterial blood gases, lung mechanics and histology, as well as to identify biological markers of inflammation and fibrogenesis in a model of ARDS with IAH.

## Methods

24 Wistar rats (250-300 g) were submitted to the a sequence of events: 1) receive *Escherichia coli* lipopolysaccharide (LPS) intraperitoneally (1,000 µg); 2) waiting period of 24 hours for development of ARDS; 3) anesthesia and mechanical ventilation; 4) induction of IAH (15 mmHg) or not; 5) random assignment to PCV (V_T_ = 6 mL/kg, respiratory rate (RR) = 80 breaths/min, fraction of inspired oxygen (FIO_2_) = 0.4 and PEEP = 5 cmH_2_O) or PSV. During PCV, animals were paralyzed with pancuronium bromide. In PCV and PSV, the driving pressure was adjusted to achieve V_T_ = 6 ml/kg. In addition, in PCV, the RR was controlled to keep minute ventilation constant (160 ml/min). Peak (Ppeak,_RS_), and mean (Pmean,_RS_) airway pressures and arterial blood gases were analyzed at baseline and at the end of 1 h ventilation. Lungs were removed for lung histology and molecular biology analysis [mRNA expression of interleukin (IL)-6, and pro-collagen type III (PCIII)].

## Results

PSV improved oxygenation regardless of IAH. In ARDS with IAH, PSV, compared to PCV group, was associated with greater reduction in Ppeak,_RS_ (PSV: 11.4 ± 2.4 cmH_2_O, PCV: 16.9 ± 0.5 cmH_2_O, p < 0.05) and Pmean,_RS_ (PSV: 5.8 ± 1.9 cmH_2_O, PCV: 9.6 ± 0.2 cmH_2_O, p < 0.05). Furthermore, PSV reduced the amount of alveolar collapse, and the mRNA expression of interleukin (IL)-6 and type III procollagen compared to PCV.

## Conclusions

In this model of ARDS with IAH, PSV, compared to PCV, promoted functional and lung morphological benefit thus mitigating VILI.

## Grant Acknowledgment

CNPq, FAPERJ, CAPES, PRONEX, INCT-INOFAR
